# Insulin-Sensitizing Properties of Decoctions from Leaves, Stems, and Roots of *Cucumis prophetarum* L.

**DOI:** 10.3390/molecules30010098

**Published:** 2024-12-30

**Authors:** Zewdie Mekonnen, Giuseppe Petito, Getasew Shitaye, Gianluca D’Abrosca, Belete Adefris Legesse, Sisay Addisu, Maurizio Ragni, Antonia Lanni, Roberto Fattorusso, Carla Isernia, Lara Comune, Simona Piccolella, Severina Pacifico, Rosalba Senese, Gaetano Malgieri, Solomon Tebeje Gizaw

**Affiliations:** 1 Department of Biochemistry, School of Medicine, College of Health Sciences, Addis Ababa University, Addis Ababa P. O. Box 9086, Ethiopia; zewdie.mekonnen@aau.edu.et (Z.M.); solomon.tebeje@aau.edu.et (S.T.G.); 2 Department of Biomedical Sciences, College of Medicine and Health Sciences, Bahir Dar University, Bahir Dar P. O. Box 79, Ethiopia; getasewshitaye.ayalew@unicampania.it; 3 Department of Environmental, Biological and Pharmaceutical Sciences and Technologies, University of Campania, 81100 Caserta, Italy; giuseppe.petito@unicampania.it (G.P.); antonia.lanni@unicampania.it (A.L.);; 4 Department of Clinical and Experimental Medicine, University of Foggia, Viale Pinto 1, 71100 Foggia, Italy; 5 Center for Innovative Drug Development and Therapeutic Trials for Africa (CDT-Africa), College of Health Sciences, Addis Ababa University, Addis Ababa P. O. Box 9086, Ethiopia; 6 Center for Study and Research on Obesity, Department of Medical Biotechnology and Translational Medicine, University of Milan, 20133 Milan, Italy; maurizio.ragni@unimi.it

**Keywords:** diabetes mellitus, medicinal plants, *Cucumis prophetarum* L., apigenin *C*-glycosides, antidiabetic effect, cytotoxicity, hyperglycemia, insulin-sensitizing properties

## Abstract

Type 2 diabetes mellitus (T2DM) is a chronic disease characterized by insulin resistance and impaired beta-cell secretory function. Since existing treatments often present side effects based on different mechanisms, alternative therapeutic options are needed. In this scenario, the present study first evaluates the cytotoxicity of decoctions from the leaves, stems, and roots of *Cucumis prophetarum* L. on HepG2 and L6C5 cells. The extracts were chemically investigated by UV–Vis and ATR-FTIR spectroscopic techniques and by ultra high-performance chromatographic techniques, coupled with high-resolution mass spectrometry. Briefly, decoctions from the leaves and stems were mainly composed of apigenin *C*-glycosides, while the root decoction was rich in raffinose and cucumegastigmane II. To evaluate the insulin-sensitizing properties of the extracts in insulin-resistant L6 myoblasts, an evaluation by Western blot analysis of the proteins in the insulin signaling pathway was then performed. Particularly, key proteins of insulin signaling were investigated, i.e., insulin receptor substrate (IRS-1), protein kinase B (PKB/AKT), and glycogen synthase kinase-3 (GSK-3β), which have gained considerable attention from scientists for the treatment of diabetes. Under all conditions tested, the three decoctions showed low cytotoxicity. The stem and root decoction (300 μg/mL) resulted in a significant increase in the levels of p-IRS-1 (Tyr612), GSK3β (Ser9), and p-AMPK (Thr172) compared to those of the palmitic acid-treated group, and the leaf decoction resulted an increase in the level of p-IRS-1 (Tyr612) and p-AMPK (Thr172) and a decrease in p-GSK3β (Ser9) compared to the levels for the palmitic acid-treated group. The root decoction also reduced the level of p-mToR (Ser2448). Overall, the acquired data demonstrate the effect of reducing insulin resistance induced by the investigated decoctions, opening new scenarios for the evaluation of these effects aimed at counteracting diabetes and related diseases in animal models.

## 1. Introduction

Type 2 diabetes mellitus (T2DM) is a metabolic disorder caused by a combination of defective insulin secretion by pancreatic β-cells and the inability of tissues to appropriately respond to insulin [1]. Accounting for 90% of diabetes cases among the world population, T2DM has been identified as a serious global health threat by World Health Organization (WHO) [2,3]. The global prevalence of impaired glucose tolerance was estimated to be 541 million (10.6%) and is projected to reach 623 million (11.0%) and 730 million (11.4%) by 2030 and 2045, respectively, which may indicate a high risk of developing type 2 diabetes [4]. According to the Global Burden of Disease Study 2021 report from an estimated 529 million people with diabetes, approximately 96.0% (508 million) had type 2 diabetes [5]. It has been also reported that in 2017, an estimated 462 million individuals were affected by T2DM, representing a significant burden to the world population, ranking it as one of leading cause of mortality [6]. Moreover, T2DM as the major diabetes case, imposes a significant economic impact on individuals and their family life, health systems, and national economies [7]. Insulin receptor (IR) and insulin receptor substrate (IRS) proteins are activated by tyrosine phosphorylation [8]. The serine phosphorylation of IR and IRS proteins impairs insulin-stimulated signaling by reducing their tyrosine phosphorylation [9,10]. Most of the roles played by insulin are mediated by signaling pathways involving IRS proteins, phosphorylation, and the activation of phosphatidylinositol-3-kinase (PI3K), protein kinase B (Akt), and the molecular target rapamycin (mTOR) [11,12]. In one of the signaling pathways for insulin action, the PI3K/AKT pathway, activation starts with the phosphorylation of the IRS-1 (Tyr612) that results in the binding and activation of the PI3K that increases the concertation of phosphatidylinositol (3,4,5)-triphosphate (PIP3), which in turn leads to the activation of PIP3-dependent kinases (PDK-1 and PDK-2), and eventually, to the activation of AKT/PKB kinase and a typical PKCs [13]. This pathway plays a role in glucose uptake and metabolic control by stimulating the translocation of GLUT-4 to the biological membrane [14]. Moreover, the activated protein kinase phosphorylates glycogen synthase kinase 3β (GSK3β) at the Ser9 residues, inactivating it, which is the key control mechanism for GSK3β activity [15]. This inactivation of GSK3β leads to the dephosphorylation and activation of glycogen synthase, which aids in glycogen synthesis [16,17]. The dysregulation of these biological cascades has been implicated in T2DM like insulin resistance condition and leads to hyperglycemia and hyperlipidemia [18,19,20]. Other signaling pathways, like AMP-activated protein kinase (AMPK), and the mammalian target of rapamycin/p70 ribosomal S6 kinase (mTOR/p70 S6K) signaling pathway was also known to affect insulin resistance [11,21,22]. The activation of AMPK requires the phosphorylation of Thr172 on the α-subunit by upstream kinases, inducing AMPK signaling activation, which is important for the alleviation of insulin resistance, as it promotes glucose entry into the cells, fatty acid oxidation, and mitochondrial biogenesis [23,24]. The phosphorylation of the mammalian target of rapamycin (mToR at Ser2448) was also linked with the condition of insulin resistance [25].

Although the first line of prevention and treatment for insulin resistance is lifestyle modification, pharmacological and non-pharmacological therapies may be necessary in many cases [26,27]. Several therapeutic options are available for T2DM, with recorded side-effects for some of these [28]. The use of medicinal plants has continued to be a good foundation of natural products for the management of different illnesses [29,30,31,32]. Thus, many decoctions from different parts of medicinal plants have been used for the management of metabolic disorders such as the decoction of *Rumex abyssinicus* root [33], *Foeniculum vulgare* leaf [34], *Moringa stenopetala* leaf [35,36], and *Cadaba farinose* root [37]. In addition, various in vitro and in vivo studies have revealed that different plant crude extracts and solvent fractions exhibit hypoglycemic effects [38,39,40]. Similarly, in Ethiopia, there are several medicinal plants used for the treatment of T2DM, and a number of these were examined for their antihyperglycemic effect [41,42].

*Cucumis prophetarum* L. is a species of tendril-bearing herb in the *Cucurbitaceae* family, in the genus *Cucumis*. It is mostly a climbing, monoecious herb with variously colored ellipsoid fruits, with green and white stripes. It is widely grown in Africa, Asia, and Australia. *C. prophetarum* has been traditionally used to treat liver and lung disorders, heart failure, diarrhea, gonorrhea, skin infections, intestinal problems, and cancer [43,44]. The health-promoting effects of the plant fruits and leaves are derived from their specialized metabolites that are able to exert antibacterial and antioxidant activities [45]. Although *C. prophetarum* fruit was investigated for its ability to inhibit α-amylase and α-glucosidase, the antidiabetic effect of other plant organs was not investigated [46,47]. In the study area where *C. prothepharum* is cultivated, traditional healer practices employ the leaf, stem, and root parts of the plant for the treatment of diabetes mellitus. In this context, this explorative study aimed to assess the efficacy of leaf, stem, and root decoctions from *C. prophetarum* using a cell model of L6 in which insulin resistance was induced. The extracts obtained, chemically profiled by means of UV, FTIR spectroscopic, and UHPLC HR-MS/MS tools, underwent in vitro evaluation to ascertain their potential beneficial effect on the insulin signaling pathway.

## 2. Materials and Methods

### 2.1. Chemicals and Reagents

The general reagents used were of the highest available grade and were obtained from Merck (St. Louis, MO, USA). Dulbecco’s Modified Eagle’s Medium (DMEM) and fetal bovine serum (FBS) were obtained from Thermo Fisher Scientific (Waltham, MA, USA). Anti-PhosphoAkt (Ser473), Anti-Akt, Anti IRS1, P-AMPKα (Thr172), AMPKα, P-mTOR (Ser2448), and mTOR were obtained from Cell Signaling (Danvers, MA, USA). Anti-P-IRS1 (Tyr612) was obtained from Invitrogen (Carlsbad, CA, USA). P-IRS1 (Ser307) was obtained from Millipore (Burlington, MA, USA). P-GSK3β (Ser9) and GSK3β (Ser9) were obtained from Abclonal (Düsseldorf, Germany). Anti-β-ACTIN antibody was obtained from BIOSS (Woburn, MA, USA). Secondary antibodies, peroxidase anti-rabbit IgG, and peroxidase anti-mouse IgG were obtained from Abcam (Cambridge, UK).

### 2.2. Plant Material Collection and Extraction

*C. prophetarum* was collected in areas around Lake Tana in the Abay River Basin in the northwest part of Ethiopia. The botanical specimen was authenticated at the National Herbarium of Addis Ababa University with the voucher specimen code ZM001. The collected plant parts (leaf, stem, and root) were washed under running tap water to remove adhering dust. The plant parts were air-dried under shade and then pulverized into coarse powder using a grinding mill. The powders obtained were packed separately in air-tight zipper bags and kept at −4 °C. After obtaining a customs permit (Ref. No: E.B.I-71/3090/2015) from Ethiopia Biodiversity Institute (EBI), a research collaboration was established after an official Memorandum of Understanding (MoU) and a Material Transfer Agreement (Ong, #5) were signed between the Department of Medical Biochemistry, Addis Ababa University, and the Department of Environmental, Biological, and Pharmaceutical Sciences and Technologies, University of Campania “Luigi Vanvitelli” (Caserta, Italy). The powders from the leaves (2.11 Kg), stems (2.15 Kg), and roots (2.15 Kg) were shipped to Caserta and were stored at −4 °C until the day of use.

The leaf, stem, and root organs of *C. prophetarum* were chemically investigated following decoction, an extraction technique useful for obtaining water-soluble substances from plant materials by boiling them in water. This technique, commonly exploited for producing self-help remedies, was also applied by traditional healers. After cooling, lyophilization was employed for preserving the extracts’ quality and extending their shelf life.

**Figure 1:**
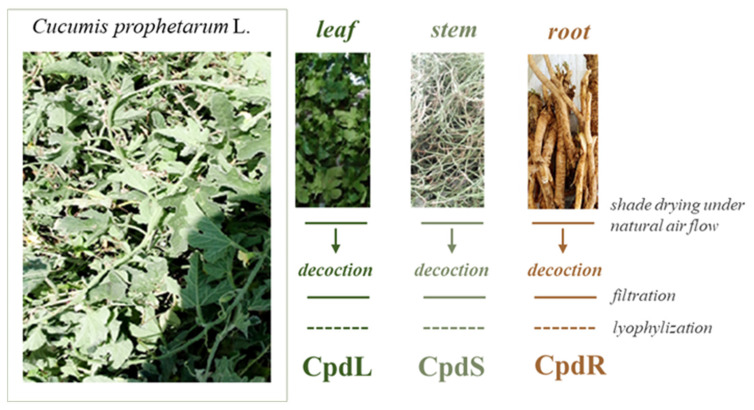
Extraction scheme applied to leaf (CpdL), stem (CpdS), and root (CpdR) organs of *Cucumis prophetarum* L. The plant parts were photographed during sample collection (around Lake Tana, Abay River Basin, northwestern Ethiopia, 2022/3).

The stem, leaf, and root powders underwent decoction, following the procedure commonly applied by traditional healers (Figure 1). To this purpose, dried powder materials were boiled in water using a plant part–water ratio equal to 1:20. The simmer time was one hour. After boiling, the mixture was cooled and filtered to separate the liquid extract from the solid residues. To achieve metabolic exhaustion of each investigated plant part, after filtration, each plant powder underwent again decoction. The filtrates obtained from the two extractions were combined, centrifuged at 2400 rpm for 5 min (Beckman Coulter Centrifuge, Milan, Italy), and lyophilized (Biocool FD-1A-50, Beijing, China). Subsequently, the freeze-dried extracts were stored at −4 °C until use.

### 2.3. UV–Vis and ATR-FTIR Spectroscopic Analyses of C. prophetarum Extracts

The *C. prophetarum* extracts were chemically analyzed by UV–Vis and attenuated total reflection (ATR)-FTIR spectroscopic tools. The UV–Vis spectra were acquired in the range of 200–800 nm using the Mobi™ Atomic Absorption Microplate Spectrophotometer (MSE Supplies, Daejeon, Republic of Korea). The IRXross Fourier-Transform InfraRed (FT-IR) spectrophotometer (Shimadzu, Milan, Italy) was utilized [wavenumber range: 4000–500 cm^−1^; resolution: 4 cm^−1^ (45 scans)] to acquire the ATR-FTIR spectra, which were further processed by using the Lab solution IR software (v. 1.60, Shimadzu, Milan, Italy).

### 2.4. UHPLC-ESI-QqTOF-MS/MS Analysis

The *C. prophetarum* decoctions were analyzed using a Shimadzu NEXERA UHPLC system, manufactured by Shimadzu (Kyoto, Japan). The separation was carried out using the Luna^®^ Omega C18 column (1.6 µm; 50 × 2.1 mm i.d; Phenomenex, Torrance, CA, USA), and a gradient elution with mobile phase sol. A (water with 0.1% HCOOH) and sol. B (acetonitrile, 0.1% HCOOH). Starting from mobile phase sol. B at 5%, it increased to 17.5% in 5 min and to 45% in 8 min. Subsequently, it held at 45% for 2 min and further increased to 95%. The system remained constant at 95% sol. B for 1 min, then the initial condition was restored, and column rebalancing was performed. The flow rate was set at 0.5 mL/min. Mass spectrometric (MS) analysis was performed using a hybrid Q-TOF MS instrument, specifically AB Sciex Triple TOF^®^ 4600 (AB Sciex, Toronto, ON, Canada), operating in negative electrospray ionization (ESI) mode. The analysis included one full scan (TOF) in the 220–1000 Da mass range and eight Information Dependent Acquisition (IDA) MS/MS scans in the 80–950 Da mass range. The parameters of the ESI source were set as follows: curtain gas, 35 psi; nebulizer gas, 60 psi; heated gas, 60 psi; ion spray voltage, 4.5 kV. Furthermore, the interface heater temperature was at 600 °C, while the declustering potential was set at −70 V. The collision energy applied was equal to −40 ± 5 V. The system was operated by Analyst^®^ TF 1.7 software, and the data were processed by PeakView^®^ version 2.2 software.

### 2.5. Cell Culture Treatment

L6 cells were cultured in DMEM supplemented with 100 U/mL penicillin, 100 U/mL streptomycin, and 10% FBS at 37 °C under a humidified atmosphere of 5% CO_2_. All cells were plated in Petri dishes for at least 48 h. The medium was replaced with 2% FBS at 80% confluence. After five days of differentiation, long tubes formed, indicating myotube formation. Subsequently, the cells were exposed for 18 h to a mixture of long-chain fatty acids (FAs) palmitate (0.75 mM final concentration) in a media with 1% BSA-free fatty acids. After 18 h of exposure, the medium was replaced with another medium containing FAs, with 300 μg/mL of aqueous extract of *C. prophetarum* leaves, stems, and roots for another 6 h. For the same period, the L6 cells were incubated without FAs. To obtain the desired final concentrations, stock solutions of 30 mM FAs were diluted in 1% BSA-containing culture medium. At the end of treatment, the cells were scraped, washed twice in sodium phosphate buffer (PBS), and stored at −80 °C until use.

### 2.6. MTT Assay

HepG2 and L6 cells were plated (100 μL/well) in triplicate in 96-multiwell plates at a density of 1.5 × 10^4^ cells/well. The day after seeding, the cells were incubated with different concentrations of decoctions of *C. prophetarum* leaves, stems, and roots. After a 24 h exposure time, the cells were treated with 150 µL of 3-(4,5-dimethyl-2-thiazolyl)-2,5-diphenyl-2H-tetrazolium (MTT; 0.5 mg/mL). The MTT solution was then removed, and 100 µL of isopropanol was added to dissolve the produced formazan dye. Finally, the absorbance at 570 nm of each sample was determined using a Sinergy H1 microplate (BioTek, Shoreline, WA, USA).

### 2.7. Oil Red O (ORO) Staining

At the end of treatment, the cells were washed two times with iced PBS 1X and fixed with 10% formalin for 10 min. Once the formalin was discarded, the cells were exposed to 10% formalin for another 1 h. After fixation, the cells were washed four times with distilled water and washed with 60% isopropanol for 5 min. The cells were stained with Oil Red O working solution for 10 min at room temperature and washed again with PBS 1X to remove unbound staining. The images were acquired under an optic microscope at 10X magnification. To quantify the Oil Red O content levels, isopropanol 60% was added to each sample.; After shaking at room temperature for 5 min, the density of the samples were read at 500 nm with a spectrophotometer.

### 2.8. Preparation of Total Lysates

Cell culture pellets were resuspended in RIPA buffer containing 150 mM NaCl, 1.0% Triton X-100, 0.5% sodium deoxycholate, 0.1% SDS, 50 mM Tris pH 8.0, 1 mM Na_3_VO_4_, 1 mM PMSF, and 1mg/mL leupeptin. To prevent the dephosphorylation of sensitive proteins during sample preparation, we used PhosSTOP™ (Roche, Basel, Switzerland).

Subsequently, the homogenate was centrifuged at 14,000× *g* for 15 min at 4 °C (Beckman Coulter S.p.A., Milan, Italy). The protein concentrations of the supernatants of the centrifuged lysates were determined using BioRad’s DC method (Bio-Rad Laboratories, s.r.l., Segrate, Italy).

### 2.9. Western Blot Analysis

Electrophoreses on SDS-PAGE gels and Western blot analysis were performed according to the methods of Petito et al. [48] and Senese et al. [49], with minor modifications. In brief, total lysates containing 15 µg protein were loaded in each lane, electrophoresed on SDS-PAGE gels, and transferred to the nitrocellulose membrane. The protein ladder used for molecular weight reference is Precision Plus Protein™ All Blue Prestained Protein Standard (1610373; Biorad) (Molecular weight 10 to 250 KDa). The membranes were blocked with 5% (*w*/*v*) nonfat dry milk (in TBS-T). The primary antibodies were diluted in TBS with 0.01% (*v*/*v*) Tween 20 (TBS-T) and 5% (*w*/*v*) bovine serum albumin (BSA), while the secondary antibodies were diluted in TBS with 0.01% (*v*/*v*) Tween 20 (TBS-T) and 5% (*w*/*v*) nonfat dry milk. The membranes were probed with the following primary antibodies: P-IRS1(Tyr612) (Invitrogen—1:1000 dilution), P-IRS1 (Ser307) (Millipore—1:500 dilution), IRS1 (Cell Signaling—1:1000 dilution), P-AKT (Ser473) (Cell Signaling—1:1000 dilution), AKT (Cell Signaling—1:1000 dilution), P-GSK3β (Ser9) (ABclonal—1:1000 dilution), GSK3β (ABclonal—1:10,000 dilution), P-AMPKα (Thr172) (Cell Signaling—1:1000 dilution), AMPKα (Cell Signaling—1:1000 dilution), P-mTOR (Ser2448) (Cell Signaling—1:1000 dilution), mTOR (Cell Signaling—1:1000 dilution), and B-ACTIN (Bioss—1:1000 dilution). As secondary antibodies, peroxidase anti-rabbit IgG (abcam—1:4000 dilution) and peroxidase anti-mouse IgG (abcam—1:4000 dilution) were used. The chemiluminescent blots were imaged using the ChemiDoc XRS+ (Biorad) and analyzed using Image Lab Software 6.0.1.

### 2.10. Ethical Consideration

Ethical clearance was obtained from the Research and Ethics Committee of the Department of Medical Biochemistry and the Institutional Review Board (IRB) of the College of Health Sciences, Addis Ababa University. The MoU, MTA, and shipping permit were obtained from Addis Ababa University and the Ethiopia Biodiversity Institute, Addis Ababa, Ethiopia. The leaf, stem, and root powders of *Cucumis prophetarum* L. were only used for the specific research collaboration under the governance of the MoU and MTA.

### 2.11. Statistical Analysis

All results were analyzed using GraphPad Prism 9 software system (GraphPad Software, San Diego, CA, USA). Data were expressed as the mean ± SEM and were normally distributed. The statistical significance of the differences between experimental groups was determined using one-way ANOVA, followed by Tukey post-hoc testing. Differences were considered statistically significant at *p* < 0.05.

## 3. Results

### 3.1. UV–Vis and ATR-FTIR Analysis of Cucumis prophetarum Extracts

**Figure 2:**
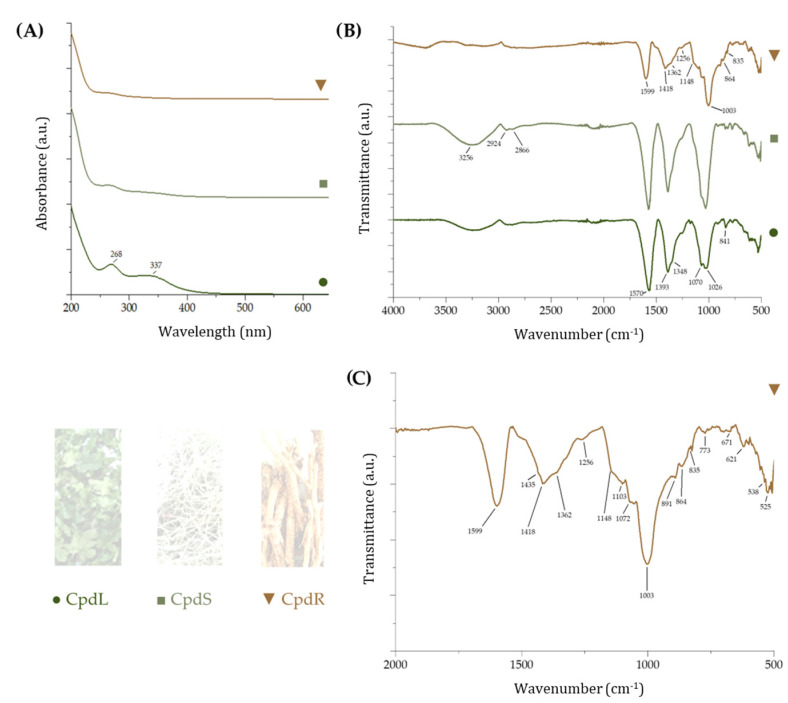
(**A**) UV–Vis spectra and (**B**) ATR-FTIR spectra of leaf decoction (CpdL), stem decoction (CpdS), and root decoction (CpdR); (**C**) enlarged region (500–2000 cm^−1^) of the ATR-FTIR spectrum of CpdR decoction.

The yield of the leaf extract (CpdL), stem extract (CpdS), and root extract (CpdR) were found to be 8.9%, 7.0%, and 5.3%, respectively. When UV–Vis spectra were acquired, it was observed that the UV spectrum of CpdL was distinguished by the abundance of bands at 337 and 268 nm, which were in line with the characteristic flavones B-ring (band I), and A-ring (band II) absorption bands. These latter were also weakly observed in the CpdS UV spectrum, while no aromatic compounds were detectable in the CpdR decoction (Figure 2A). Analogously, for the ATR-FTIR spectra of the CpdL and CpdS extracts, a band at 2970 cm^−1^ was detected, beyond a broad band, at wavenumbers 3000–3600 cm^−1^, which was due to the OH stretching vibrations and the intermolecular hydrogen bonds. This finding suggested the C–H stretching vibration of flavone *C*-glycosides, previously isolated from leaves of other *Cucumis* spp. [50], while the band at 2920(4) cm^−1^ was assignable to aliphatic C–H stretching vibrations. The flavonoid occurrence was further highlighted through the C=C stretching vibrations at 1650–1560 cm^−1^ and at 1380–1350 cm^−1^. The latter were commonly assigned to the C=C bonds in flavones [51] (Figure 2B).

The ATR-FTIR spectra of the CpdR extract suggested saccharide occurrence. In particular, in region 500–2000 cm^−1^, the spectra displayed, beyond the C–H and C–O in-plane bending vibrations, were detectable at 1435, 1418 cm^−1^, and 1256 cm^−1^. The latter was assigned to the C–H in plane bending mode of anomeric carbon. Moreover, the bands at 1003 and 1148 cm^−1^ were due to the CO stretching of the α-(1,6)-glycosidic bond [52]. Furthermore, the band at 864 cm^−1^ was in line with the furanose ring vibration, while that at 835 cm^−1^ was attributable to the α-glycosidic bond occurrence, beyond the deformation bands at 538 (due to the glucose ring), 621, and 671 cm^−1^ (attributable to the galactose ring). These data were in line with raffinose occurrence. This trisaccharide, which is comprised of α-d-galactose, α-d-glucose, and β-d-fructose, was commonly found in cucurbits, together with stachyose and galactinol [53].

### 3.2. UHPLC-ESI-QqTOF-HR MS/MS Analyses of C. prophetarum Extracts

**Figure 3:**
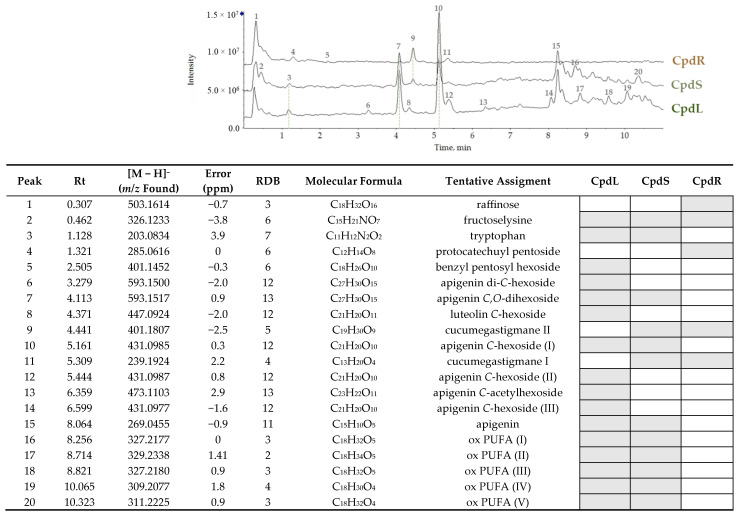
Total ion current (TIC) chromatograms of leaf, stem, and root extracts obtained by decoction. TOF-MS data are tabulated below, along with ring double bonds (RDB) data. Ox PUFA = oxidized polyunsaturated fatty acids.

To gain insight into the chemical composition of the decoction extracts from the leaves, stems, and roots of *C. prophetarum*, UHPLC-ESI-QqTOF HR MS/MS analyses were carried out. The total ion current chromatograms and the TOF-MS data are reported in Figure 3, while the TOF-MS/MS spectra of the tentatively assigned compounds are included in the Supplementary Materials. It appeared evident that the highest metabolic diversity belonged to the CpdL and CpdS extracts, while the root decoction consisted of only a few compounds. In fact, in the CpdR extract, according to ATR-FTIR data, a trisaccharide was detected and likely identified as raffinose. The TOF-MS spectrum of the compound showed the deprotonated molecular ion at *m*/*z* 503.1614, while its chloride adduct at *m*/*z* 539.1391 was also observed. The [M − H]^−^ ion gave rise in the TOF-MS/MS experiment (Figure S1) to predominant ions at *m*/*z* 221.0673 and 179.0582, belonging to raffinose [54] Cucumegastigmane II (**9**) and cucumegastigmane I (**11**), previously isolated from the leaves of *Cucumis sativus* [55], and recently reported as carotenoid by-products in different organs of *Cucumis melo* var. *flexuosus* [56], were also identified as abundant compounds in CpdR extract. The [M − H]^−^ ion of cucumegastigmane II (**9**) at *m*/*z* 401.1807 provided the TOF-MS/MS ion at *m*/*z* 221.1185, which occurred through the neutral loss of the hexose moiety (Figure S1). Finally, the root decoction contained a pentosyl derivative of protocatechuic acid (**4**), which exhibited the TOF-MS deprotonated ion at *m*/*z* 285.0616. The latter underwent a −132.04 Da neutral loss, as noted by the (pentose-H_2_O) residue (Figure S1).

Glycosylated flavones were found in both leaf and stem extracts, which also shared fructoselysine (**2**) and tryptophan (**3**). Benzyl pentosyl hexoside, with the [M − H]^−^ ion at *m*/*z* 401.1452, was also weakly observed in the leaf extract. The most abundant compounds are apigenin glycosides, whose TOF-MS/MS spectra, together with that of the only detected luteolin glycoside, are reported in Figure S2. Briefly, two isomers, sharing the [M − H]^−^ ion at 593.15, were detected [57]. The first one, which was found only in the leaf extract, was tentatively identified as apigenin 6,8-di-*C*-hexoside, while the more abundant second one was identified as apigenin-6-*C*-hexoside-7-*O*-hexoside. The deprotonated molecular ion of compound **7** provided TOF-MS/MS fragment ions at at *m*/*z* 473.1085 through neutral loss of 120.05 Da due to cross-ring cleavage in *C*-bonded hexose, and at *m*/*z* 431.0972, following the loss of a dehydrated hexose moiety. Furthermore, three isomers (**10**, **12**, and **14**) with the deprotonated molecular ion at *m*/*z* 431.10 were noticed. The [M − H]^−^ ion of compound **10**, which was in both CpdL and CpdS extracts, underwent cross-ring cleavages to give the ions at *m*/*z* 341.0667 (−90.03 Da) and 311.0551 (−120.05 Da). The compound was tentatively identified as apigenin 6-*C*-hexoside. A similar fragmentation occurred for compounds **12** and **14**, which were only in leaf decoction, together with luteolin *C*-hexoside (**8**). In particular, compounds **12** and **14** were hypothesized to be apigenin 8-*C*-hexosides, differing each other for the hexosyl moiety identity. Furthermore, both the extracts accounted for trihydroxyoctadecadienoic acid with [M − H]^−^ ion at *m*/*z* 327.2170 [58,59]. Finally, the [M − H]^−^ ion of compound **13** was at *m*/*z* 473.1103 was attributable to apigenin-6-*C*-acetylhexoside. The TOF-MS/MS experiment showed the neutral loss of the acetic acid (−60.02 Da) due to the occurrence of an acetyl moiety on the apigenin 6-*C*-hexoside skeleton. Apigenin aglycone (**15**), with a deprotonated molecular ion at *m*/*z* 269.0455, was also found in the leaf and stem decoctions. Furthermore, the leaf and stem extracts contained differently oxidized unsaturated fatty acids, with eighteen carbon atoms (**17**–**20**; Figure S3) [60].

### 3.3. Cytotoxicity Screening of Decoction Extracts from C. prophetarum Organs

**Figure 4:**
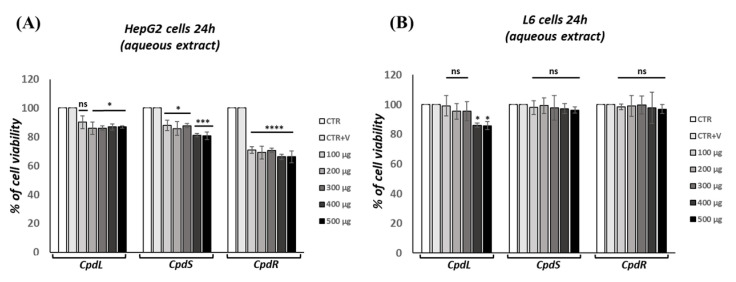
Cell viability evaluation. The viability of (**A**) HepG2 and (**B**) L6 cells was analyzed after 24 h for leaf, stem, and root decoction exposition compared to the results for the control (100%). Data are represented as mean ± SEM of the percentage of viable cells compared to that of the CTR group. * *p* < 0.05; *** *p* = 0.0001; **** *p* < 0.0001. ns = not significant.

The cytotoxicity of *C. prophetarum* organs was assessed in the HepG2 and L6 cell lines by MTT assay, which is widely used in the in vitro evaluation of the biosafety of plant extracts [3,20,28]. The viability of HepG2 cells after 24 h of exposure to leaf, stem, and root decoction extracts at concentrations ranging from 100 to 500 µg/mL was between 70% and 90% (Figure 4A). L6 cell viability was not affected by leaf, stem, and root decoction extracts at different concentrations. Only the leaf at 400 and 500 µg/mL exhibited a decrease in cell viability after 24 h of exposure time (Figure 4B). In all performed experiments, the vehicle application did not interfere with cell viability. We chose to use 300 µg/mL for further evaluations. In the L6 cells, this dosage represents the highest dose at which none of the decoction extracts exhibited any effects on cell viability.

### 3.4. Effect of Decoction Extracts from C. prophetarum on the Lipid Accumulation in L6 Skeletal Muscle Cells Exposed to Palmitic Acid

**Figure 5:**
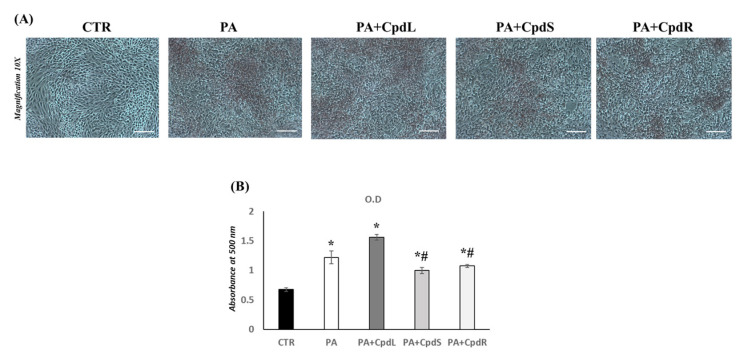
(**A**,**B**) Oil Red O staining and Oil Red O quantification (OD value) of L6 skeletal muscle cells treated with PA (0.75 mM), PA and a decoction extract of leaves, stems, and roots (300 μg/mL) of *C. prophetarum.* Cells were observed under 10X magnification, with a scale bar of 2 μm. Values are represented as means ± SEM; (n = 3/group). One-way ANOVA was used for statistical analysis. *p* < 0.05 was considered significant. * *p* < 0.05 vs. CTR; # *p* < 0.05 vs. PA. CTR: control; PA: palmitic acid; PA + CpdL: palmitic acid plus leaf decoction extract; PA + CpdS: palmitic acid plus stem decoction extract; PA + CpdR: palmitic acid plus root decoction extract.

Firstly, we have verified that treatment with palmitic acid was able to induce fat accumulation in L6 cells. In Figure 5, the ORO staining and ORO semi-quantification values of the L6 cells exposed to palmitic acid and leaf, stem, and root decoction extract (300 μg/mL) of *C. prophetarum* are shown. The results revealed a significantly increase in lipid accumulation in L6 skeletal muscle cells treated with palmitic acid compared to the results for the CTR group (Figure 5A,B). Also, in PA + CpdL, we have observed an increase in lipid accumulation when compared to that of the CTR group (Figure 5A). The PA + CpdS and PA + CpdR groups showed a significant reduction in lipid accumulation compared to that of the PA group (Figure 5A,B).

### 3.5. Effect of Decoction Extract from C. prophetarum on the Insulin Signaling Pathway in Palmitic Acid-Induced Insulin Resistance L6 Cells

**Figure 6:**
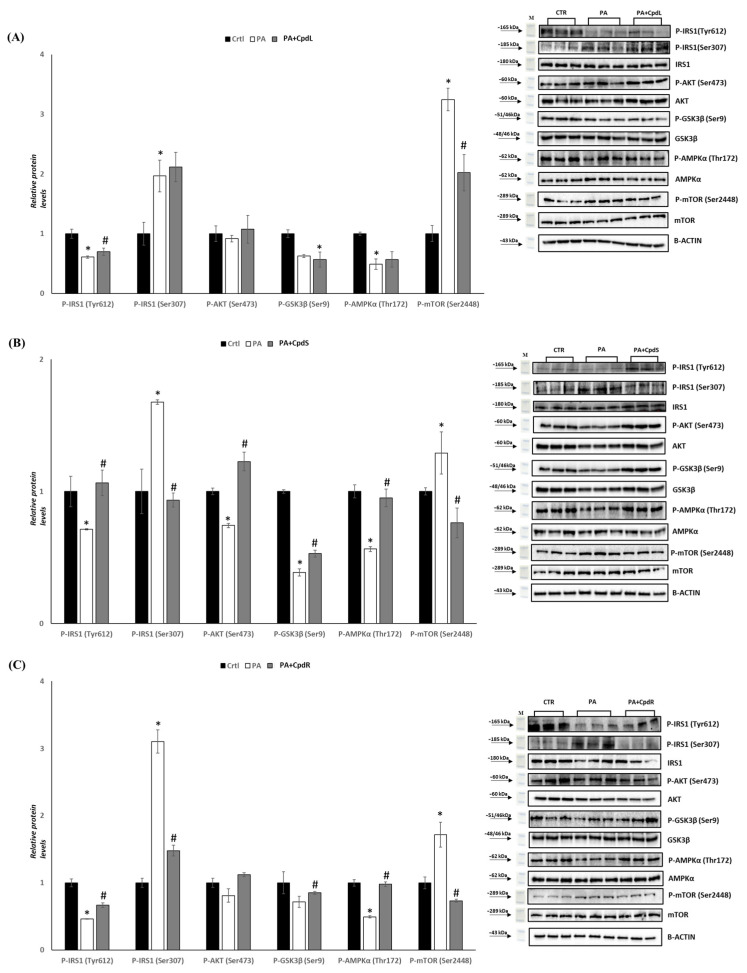
Effect of leaf, stem, and root decoction extract from *C. prophetarum* on insulin signaling molecules. L6 cells were pretreated with PA (0.75 mM) for 18 h, followed by treatment with leaf, stem, and root decoction extract of *C. prophetarum* for 6 h. (**A**–**C**) Representative immunoblots of P-IRS1 (Tyr612), P-IRS1 (Ser307), P-AKT (Ser473), P-GSK3β (Ser9), P-AMPKα (Thr172), and P-mTOR (Ser2448). Histograms show the results of the densitometric analysis of immunoblots. B-ACTIN was used as a loading control. Data are represented as mean ± SEM; n = 3. * *p* < 0.05 vs. CTR; # *p* < 0.05 vs. PA (one-way ANOVA). **CTR:** control; **PA:** palmitic acid; **PA + CpdL:** palmitic acid plus leaf decoction extract; **PA + CpdS:** palmitic acid plus stem decoction extract; **PA + CpdR:** palmitic acid plus root decoction extract.

Skeletal muscle plays a physiologically pivotal role in glucose metabolism. It absorbs 70–90% of glucose from the blood in healthy individuals [61]. To investigate whether leaf, stem, and root decoction extracts at 300 μg/mL have a protective effect on palmitic acid (PA)-induced defects in insulin signaling pathways, the phosphorylation levels of key protein molecules in the insulin signaling pathway, such as insulin-stimulated insulin receptor substrate (IRS)-1 Tyr(612), IRS-1 Ser(307), AKT Ser(473), glycogen synthase kinase-3β Ser(9), AMP-activated protein kinase Thr(172), and the mammalian target of rapamycin Ser(2448), were analyzed by Western blotting. To induce insulin resistance, an in vitro model of L6 cells treated with palmitic acid (PA) at 0.75 mM was used [62]. Exposure of L6 skeletal muscle cells to PA resulted in a significant decrease (*p* < 0.05) in the Tyr612 phosphorylation of IRS1 compared to that of the control group. Furthermore, in the presence of leaf, stem, and root decoction extracts, P-IRS1 (Tyr612) was significantly increased compared to that in the PA-treated group (Figure 6A–C). To better analyze the insulin signaling pathway, the Ser307 phosphorylation of IRS1 was measured. It is well established that the phosphorylation of Ser307 correlates with insulin resistance [63,64]. Our results revealed that in the PA group, the Ser307 phosphorylation of IRS1 was markedly increased compared to that of the control group (Figure 6A–C). However, in the PA + CpdS and PA + CpdR groups, a significant decrease in Ser307 phosphorylation levels was observed compared to that of the PA group (Figure 6B,C). Regarding the AKT phosphorylation levels, they were unchanged in the PA + CpdL and PA + CpdR groups compared to those of the PA group (Figure 6A,C). Instead, AKT phosphorylation levels were significantly increased in PA + CpdS compared to the PA group (Figure 6B). The effect of leaf, stem, and root decoction extracts of *C. prophetarum* on GSK3β (Ser9) was also examined. As shown in Figure 6, P-GSK3β (Ser9) levels were decreased in the PA group compared to those of the control group. However, in the PA + CpdS and PA + CpdR groups, a significant increase in GSK3β phosphorylation levels was observed in comparison to those for PA alone (Figure 6B,C). In addition, AMPK and mTOR phosphorylation were analyzed to investigate the effect of leaf, stem, and root decoction extracts on palmitate-induced insulin resistance in L6 cells. The P-AMPKα (Thr172) level was significantly decreased (*p* < 0.05) in the PA group compared to that of the control, whereas in the PA + CpdS and PA + CpdR groups, it was significantly increased compared to that of the PA group (Figure 6A,C). The data acquired also showed a significant increase in P-mTOR (Ser2448) levels in the PA group compared to those of the control group (Figure 6). Furthermore, in the PA + CpdL, PA + CpdS, and PA + CpdR groups, there was a significant decrease in mTOR phosphorylation levels compared to those in the PA group (Figure 6A–C).

## 4. Discussion

T2DM arises when the body cannot produce enough insulin or use it properly. This pathology causes hyperglycemia as a primary manifestation and ranks as one of the fastest-growing global health emergencies of this century [65]. T2DM is typically treated with diet, moderate exercise, and hypoglycemic and lipid-lowering drugs. Although most T2DM medications have therapeutic benefits, they can also produce undesirable side effects [28]. The discovery of natural products has become a significant resource for T2DM treatment. Several in vitro and in vivo studies have revealed that crude extracts and solvent fractions of different plant parts have anti-hyperglycemic effects and can be used to treat insulin resistance [38,39,40]. Using streptozotocin-nicotinamide-induced type 2 diabetic rats, Kavishankar and Lakshmidevi found that *N*-trisaccharide, an active compound isolated from *C. prophetarum*, is beneficial for diabetes management [66]. In 2014, the same laboratory demonstrated that a water-soluble fraction of *C. prophetarum* fruit showed potent antidiabetic and antioxidant activities [67]. Scientific research uses several in vitro models to study insulin resistance and the beneficial effects of natural products on this disorder. The C2C12 and L6 cell lines are mainly used, as they maintain the insulin signaling pathways such as IRS1, PI3K, AMPK, mTOR, and AKT [68].

The L6 rat skeletal muscle cell is a well-established model for studying glucose metabolism and mitochondrial function [69,70]. Although it is known that *C. prophetarum* fruits exert antidiabetic and antioxidant effects, no studies have been performed to determine whether its leaf, stem, and root extracts affect insulin signaling. In light of the above, this explorative study aimed to assess the efficacy of plant extracts from *C. prophetarum* using an L6 cell model in which insulin resistance was induced. Excess amounts of PA were reported to damage the mitochondria, increase oxidative stress, promote endoplasmic reticulum (ER) stress, and subsequently, elicit insulin resistance in the hepatocytes and muscle cells [71]. In the current study, the condition of insulin resistance was created by treating the L6 myotube with PA (0.75 mM) [72]. When treatment was carried out on HepG2 cells using the decoction extracts of *C. prophetarum*, the viability of the HepG2 cells after 24 h of exposure to leaf, stem, and root extracts at concentrations from 100 to 500 µg/mL was between 70% and 90% (Figure 4). Cell viability was comparable to that of the control group in the L6 cells exposed to leaf, stem, and root decoction extracts.

The skeletal muscle uptakes and processes 80% of the postprandial glucose, indicating that it plays a crucial role in regulating systemic glucose levels [69]. Both impairments in insulin signaling and the dysfunction of β-cells can lead to insulin resistance, metabolic syndrome, and T2DM [73]. In addition, mechanistically, the intracellular lipid accumulation in the skeletal muscle and the liver is a primary metabolic aberration underpinning insulin resistance [74]. Serine/threonine residue phosphorylation of insulin receptor substrates (IRS1 Ser307) instead of tyrosine phosphorylation (Tyr612) causes insulin resistance [75,76,77,78]. This alters the downstream effector Akt and could decrease glucose transporter 4 (GLUT4) translocation, reducing glucose uptake. In addition, Akt inhibits GSK3, leading to glycogen synthesis [79,80]. A decrease in IRS1_Tyr612_ and AKT_Ser473_ phosphorylation leads to insulin resistance and hyperglycemia [81]. The present study examined the insulin-sensitizing properties of decoction extracts of *C. prophetarum* in L6 myotubes in which insulin resistance was induced by PA (0.75 mM) [71,72]. Our data showed that palmitic acid treatment was able to induce lipid accumulation in L6 cells. Leaf decoction extract was ineffective to counteract lipid accumulation in cells exposed to fatty acid, while stem and root decoction extracts were able to reduce lipid accumulation in cells treated with palmitic acid (Figure 5). The levels of insulin signaling proteins (IRS1_Tyr612_, IRS1_Ser307_, AKT_Ser473_, GSK3β_Ser9_, AMPKα_Thr172_, and mTOR_Ser2448_) were measured to observe the antidiabetic effect on the alleviation of insulin resistance. Through measurement of the levels of insulin signaling proteins, the data acquired showed that L6 myotubes exposed to leaf decoction extract (300 μg/mL) for 6 h significantly (*p* < 0.05) increased the level of P-IRS1 (Tyr612) compared to that of the PA-treated group. Although the leaf decoction extract seemed to stimulate an increase in the level of P-AKT (Ser473) and P-GSK3β (Ser9), the level of P-IRS1 (Ser307) was slightly higher than that in the PA-treated group (Figure 6A). These data agree with the ineffective action shown by the leaf decoction extract on lipid accumulation in L6 cells exposed to palmitic acid. The observed increase in the level of the P-IRS1 (Ser307) indicates that leaf decoction extract triggers the insulin signaling pathway, although the downstream targets are not yet activated. The levels of P-IRS1 (Tyr612) P-AKT (Ser473) and P-GSK3β (Ser9) were significantly increased (*p* < 0.05) in the stem decoction extract (300 μg/mL) treated group compared to those in the PA-treated group (Figure 6B). Similarly, the root decoction extract resulted in a significant (*p* < 0.05) increase in the level of P-IRS1 (Tyr612) and P-GSK3β (Ser9) compared to that in the PA-treated group. The level of P-AKT (Ser473) was slightly higher compared to that in the PA-treated group. A significant (*p* < 0.05) decrease in the level of P-IRS1 (Ser307) was also observed compared to that in the PA-treated group (Figure 6C). Previous findings regarding raffinose, which is one of the main constituents of root decoction extract, highlighted its ability to increase glucose uptake in a dose-dependent manner, while it enhanced insulin sensitivity by promoting an increase in GLUT4 translocation via IRβ/PI3K/Akt phosphorylation. Furthermore, raffinose was found to inhibit the activation of GSK3 for glycogen synthesis [82].

The IRS-1/PI3K/AKT is a classic insulin signaling pathway that modulates glucose transport by activating insulin in the skeletal muscles [19,20]. The observed induction in the phosphorylation level of the insulin-signaling protein by the decoction extracts in the insulin-resistant L6 myotubes suggested that *C. prophetarum* can improve insulin resistance in T2DM. In addition, the increase in the phosphorylation level of these proteins suggested that the insulin resistance alleviation effect of the plant extract is obtained by stimulating the IRS1/PI3K/AKT signaling pathway. In line with the current study, different plant metabolites were reported to alleviate insulin resistance by regulating the IRS1/PI3K/AKT signaling pathway [77,79,83].

Insulin resistance is also affected through AMPK and the mammalian target of the rapamycin/p70 ribosomal S6 kinase (mTOR/p70 S6K) signaling pathways [11,21,22]. In skeletal muscles, AMPK activation stimulates glucose uptake, FA oxidation, GLUT4 translocation, and mitochondrial biogenesis, while inhibiting protein and glycogen synthesis [23,24]. Activation of AMPK is associated with reduced inhibitory phosphorylation of IRS1/2, ER stress, fatty acid synthase, and chronic inflammation that improves insulin sensitivity. The activation of AMPK is phosphorylated on the α-subunit activation loop at Thr172, which is the most potent activator of AMPK, increasing its activity greater than 100-fold. Activation of AMPK inhibits the mammalian targets of rapamycin complex 1 (mTORC1) [84]. mToRC1 hyperactivity elicits insulin resistance via ER stress, which induces reactive oxygen species and chronic inflammation [25].

In our study, the PA-treated group showed a high level of P-mTOR (Ser2448) and a lower level of P-AMPKα (Thr172). The level of P-AMPKα (Thr172) was significantly increased by exposure to the insulin-resistant L6 myotube, with the stem and root decoction extracts accompanied by a low level of P-mTOR (Ser2448) (*p* < 0.05). In addition, the leaf decoction extract resulted in a significant decrease in the level of P-mTOR (Ser2448), although there is a slight increase in the level of AMPK. The observed results revealed that the plant extract could exert an insulin-resistance alleviation effect through the impairment of the mTOR/p70 S6K signaling pathway. A study carried out in obese and type 2 diabetic rodents revealed that increased mTOR/p70 S6K signaling contributed to insulin resistance [85]. Reduced AMPK activity allows for the activation of mTOR, which, in turn, contributes to insulin resistance [86,87,88]. These results indicated that the activation of AMPKα leads to the inactivation of mTOR.

Specialized metabolites, such as flavonoids, as well as oligo- and polysaccharides from different plants, showed antidiabetic effects by improving insulin sensitivity [89].

Apigenin *C*-glycosides, such as vitexin and isovitexin, were found to exert promising antidiabetic effects by inhibiting RLAR, HRAR, AGEs formation, and PTP1B [90]. A recent review shows that vitexin (apigenin-8-*C*-glucoside) exerts its action on multiple targets to attenuate diabetes mellitus and its complications [81]. In fact, vitexin is protective under conditions of adipose tissue dysfunction, impaired sexuality and fertility, pancreatic β-cell dysfunction, hyperglycemia, diabetic neuropathy, and liver disorders. Beneficial effects have also been observed in regards to diabetic nephropathy, vascular disease, platelet aggregation, and hypertension [91].

Moreover, several in vitro studies evidenced that polyphenols, pure or in mixtures, exert antidiabetic activity [38,39,40], while a novel *N*-trisaccharide (containing two hexose monosaccharides and one inositol), isolated from the aqueous fruit extract of *C. prophetarum* species in India, was reported to exhibit antidiabetic properties [66]. Although abiotic factors, such as soil nature and climate, can influence the full expressive metabolic potential of the plants in terms of quality and quantity during their entire growth period (germination, growth, and maturity), the observed antidiabetic effect of the investigated *C. prophetarum* plant decoction extracts can be diversely attributed to the identified flavonoid *C*-glycosides, cucurbitacin, and raffinose.

## 5. Conclusions

The current study demonstrated that *C. prophetarum* exhibits a promising antidiabetic effect, which is expressed through the preparation of decoctions, as a usual practice of natural healers. It was observed that the extracts activate key proteins in the insulin signaling pathway, suggesting that the plant could have an insulin-resistance alleviating effect by stimulating the IRS1/PI3K/AKT signaling pathway and controlling the phosphorylation of P-mTOR and P-AMPKα. The decoctions are non-cytotoxic and show a higher antidiabetic potential, particularly those obtained from the roots, which are rich in raffinose. The acquired data lay the foundation for further studies aimed at isolating the most abundant specialized metabolites, herein tentatively identified in mixtures, and evaluating the antidiabetic effect of the extracts and pure compounds using animal models.

## Data Availability

The data supporting this article have been included as part of the Supplementary Information.

## Supplementary Materials

The following supporting information can be downloaded at: https://www.mdpi.com/article/10.3390/molecules30010098/s1, Figure S1. TOF-MS/MS spectra of compounds **1**, **4**, **9**, and **11** in CpdR extract; Figure S2. TOF-MS/MS spectra of tentatively identified apigenin glycosides, luteolin *C*-hexoside (**8**), and apigenin aglycone (**15**); Figure S3. TOF-MS/MS spectra of the detected oxidized fatty acids.
