# Supplementary material for: Insulin-Sensitizing Properties of Decoctions from Leaves, Stems, and Roots of Cucumis prophetarum L
Source: Molecules. 2024 Dec 30;30(1):98. doi: 10.3390/molecules30010098 (PMC11722063; doi:10.3390/molecules30010098)
Supplement: Supplementary file 1 [file molecules-30-00098-s001.zip › molecules-3345490-supplementary.pdf]

Insulin-Sensitizing Properties of Decoctions from Leaves, Stems, and Roots of *Cucumis prophetarum* L.

Zewdie Mekonnen [1,2,†], Giuseppe Petito [3,†], Getasew Shitaye [2,3], Gianluca D'Abrosca [4], Belete Adefris Legesse [5], Sisay Addisu [1], Maurizio Ragni [6], Antonia Lanni [3], Roberto Fattorusso [3], Carla Isernia [3], Lara Comune [3], Simona Piccolella [3], Severina Pacifico [3], Rosalba Senese [3,*], Gaetano Malgieri [3,*] and Solomon Tebeje Gizaw [1]

[1] Department of Biochemistry, School of Medicine, College of Health Sciences, Addis Ababa University, Addis Ababa P. O. Box 9086, Ethiopia; zewdie.mekonnen@aau.edu.et (Z.M.); solomon.tebeje@aau.edu.et (S.T.G.)
[2] Department of Biomedical Sciences, College of Medicine and Health Sciences, Bahir Dar University, Bahir Dar P. O. Box 79, Ethiopia; getasewshitaye.ayalew@unicampania.it
[3] Department of Environmental, Biological and Pharmaceutical Sciences and Technologies, University of Campania, 81100 Caserta, Italy; giuseppe.petito@unicampania.it (G.P.); antonia.lanni@unicampania.it (A.L.)
[4] Department of Clinical and Experimental Medicine, University of Foggia, Viale Pinto 1, 71100 Foggia, Italy
[5] Center for Innovative Drug Development and Therapeutic Trials for Africa (CDT-Africa), College of Health Sciences, Addis Ababa University, Addis Ababa P. O. Box 9086, Ethiopia
[6] Center for Study and Research on Obesity, Department of Medical Biotechnology and Translational Medicine, University of Milan, 20133 Milan, Italy; maurizio.ragni@unimi.it
* Correspondence: rosalba.senese@unicampania.it (R.S.); gaetano.malgieri@unicampania.it (G.M.)
† These authors contributed equally to this work.

Supplementary materials

1

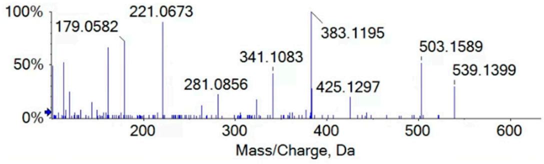

4

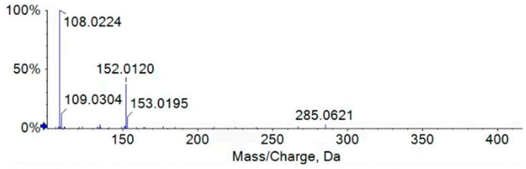

9

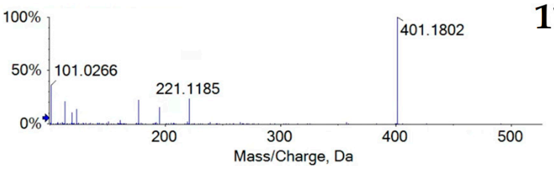

11

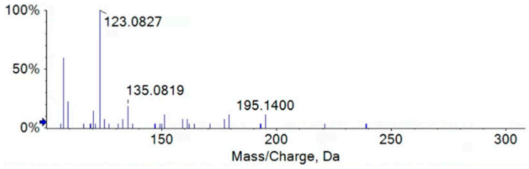

Figure S1. TOF-MS/MS spectra of compounds **1**,**4**,**9**, and **11** in CpdR extract.

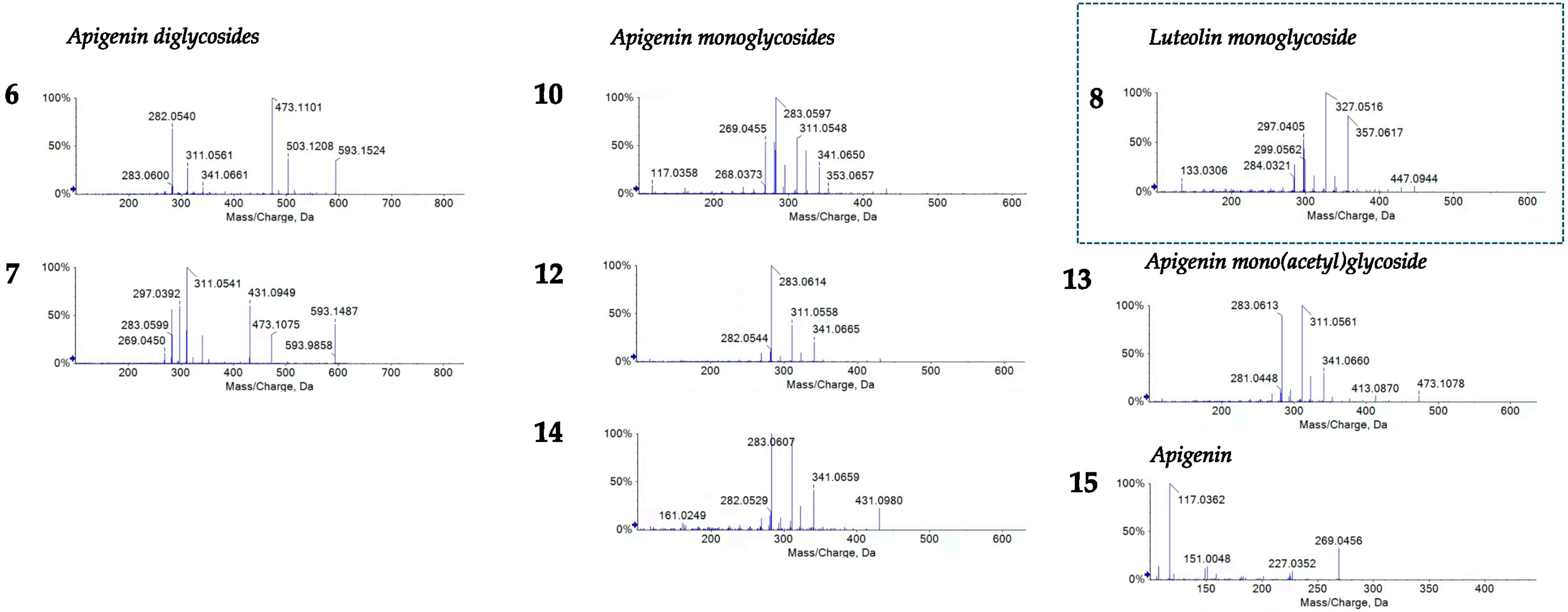

Figure S2. TOF-MS/MS spectra of tentatively identified apigenin glycosides, luteolin *C*-hexoside (**8**), and apigenin aglycone (**15**).

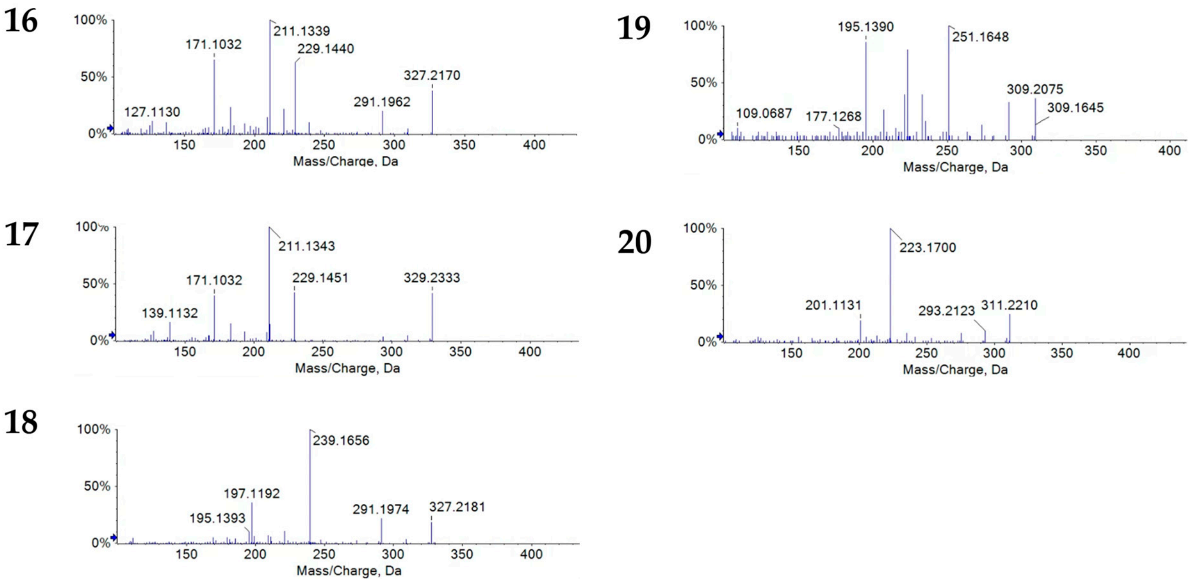

Figure S3. TOF-MS/MS spectra of the detected oxidized fatty acids.
